# Supplementary material for: End Sequence Analysis Toolkit (ESAT) expands the extractable information from single-cell RNA-seq data
Source: Genome Res. 2016 Oct;26(10):1397–410. doi: 10.1101/gr.207902.116 (PMC5052061; doi:10.1101/gr.207902.116)
Supplement: Supplemental Material [file supp_gr.207902.116_Supp_Materials.tar.gz › ESAT-esat_v0.1/doc/html/classbroad_1_1core_1_1util_1_1_parse_g_c_t_file.html]

ESAT: broad.core.util.ParseGCTFile Class Reference


ESAT


- Main Page
- Related Pages
- Packages
- Classes
- Files

- Class List
- Class Index
- Class Hierarchy
- Class Members

All Classes Namespaces Files Functions Variables Enumerator Pages

- broad
- core
- util
- ParseGCTFile

Static Public Member Functions |
List of all members

broad.core.util.ParseGCTFile Class Reference

|  |  |
| --- | --- |
| Static Public Member Functions | |
| static Map | loadData (File file) throws IOException |
|  | |
| static Map< String, String > | loadPIDName (File file) throws IOException |
|  | |
| static Map< String, ArrayList > | parseCDT (File file) throws IOException |
|  | |
| static Map< String, String > | parseChipFile (File file) throws IOException |
|  | |
| static ArrayList< String > | getOrderedListCDT (File file) throws IOException |
|  | |
| static void | writeGCT (String save, Map< String, ArrayList > gct) throws IOException |
|  | |
| static ArrayList | parseCLS (File file) throws IOException |
|  | |
| static String | getControlClass (File file) throws IOException |
|  | |
| static Set | parseCLSGroups (File file) throws IOException |
|  | |
| static Map | getGroupIndexes (ArrayList< String > groups) |
|  | |
| static Map< String, ArrayList > | getGroupIndexes (File clsFile) throws IOException |
|  | |
| static Map< Integer, String > | getIndexGroup (ArrayList< String > groups) |
|  | |
| static void | writeGMTFile (String save, Map< String, Collection< String >> gmtFile) throws IOException |
|  | |
| static MatrixWithHeaders | parseNumericCls (File file, List< String > header) throws NumberFormatException, IOException |
|  | |
| static Collection< String > | parseGeneList (File file) throws IOException |
|  | |
| static Map< String, ArrayList  < Double > > | logTransform (Map< String, ArrayList< Double >> data, double addFactor) |
|  | |

## Member Function Documentation

|  |  |  |  |  |  |
| --- | --- | --- | --- | --- | --- |
| static String broad.core.util.ParseGCTFile.getControlClass | ( | File | *file* | ) | throws IOException |

static

|  |  |  |  |  |  |
| --- | --- | --- | --- | --- | --- |
| static Map broad.core.util.ParseGCTFile.getGroupIndexes | ( | ArrayList< String > | *groups* | ) |  |

static

|  |  |  |  |  |  |
| --- | --- | --- | --- | --- | --- |
| static Map<String, ArrayList> broad.core.util.ParseGCTFile.getGroupIndexes | ( | File | *clsFile* | ) | throws IOException |

static

Here is the call graph for this function:

|  |  |  |  |  |  |
| --- | --- | --- | --- | --- | --- |
| static Map<Integer, String> broad.core.util.ParseGCTFile.getIndexGroup | ( | ArrayList< String > | *groups* | ) |  |

static

|  |  |  |  |  |  |
| --- | --- | --- | --- | --- | --- |
| static ArrayList<String> broad.core.util.ParseGCTFile.getOrderedListCDT | ( | File | *file* | ) | throws IOException |

static

|  |  |  |  |  |  |
| --- | --- | --- | --- | --- | --- |
| static Map broad.core.util.ParseGCTFile.loadData | ( | File | *file* | ) | throws IOException |

static

|  |  |  |  |  |  |
| --- | --- | --- | --- | --- | --- |
| static Map<String, String> broad.core.util.ParseGCTFile.loadPIDName | ( | File | *file* | ) | throws IOException |

static

|  |  |  |  |
| --- | --- | --- | --- |
| static Map<String, ArrayList<Double> > broad.core.util.ParseGCTFile.logTransform | ( | Map< String, ArrayList< Double >> | *data*, |
|  |  | double | *addFactor* |
|  | ) |  |  |

static

|  |  |  |  |  |  |
| --- | --- | --- | --- | --- | --- |
| static Map<String, ArrayList> broad.core.util.ParseGCTFile.parseCDT | ( | File | *file* | ) | throws IOException |

static

|  |  |  |  |  |  |
| --- | --- | --- | --- | --- | --- |
| static Map<String, String> broad.core.util.ParseGCTFile.parseChipFile | ( | File | *file* | ) | throws IOException |

static

|  |  |  |  |  |  |
| --- | --- | --- | --- | --- | --- |
| static ArrayList broad.core.util.ParseGCTFile.parseCLS | ( | File | *file* | ) | throws IOException |

static

Here is the caller graph for this function:

|  |  |  |  |  |  |
| --- | --- | --- | --- | --- | --- |
| static Set broad.core.util.ParseGCTFile.parseCLSGroups | ( | File | *file* | ) | throws IOException |

static

|  |  |  |  |  |  |
| --- | --- | --- | --- | --- | --- |
| static Collection<String> broad.core.util.ParseGCTFile.parseGeneList | ( | File | *file* | ) | throws IOException |

static

|  |  |  |  |
| --- | --- | --- | --- |
| static MatrixWithHeaders broad.core.util.ParseGCTFile.parseNumericCls | ( | File | *file*, |
|  |  | List< String > | *header* |
|  | ) |  | throws NumberFormatException, IOException |

static

|  |  |  |  |
| --- | --- | --- | --- |
| static void broad.core.util.ParseGCTFile.writeGCT | ( | String | *save*, |
|  |  | Map< String, ArrayList > | *gct* |
|  | ) |  | throws IOException |

static

|  |  |  |  |
| --- | --- | --- | --- |
| static void broad.core.util.ParseGCTFile.writeGMTFile | ( | String | *save*, |
|  |  | Map< String, Collection< String >> | *gmtFile* |
|  | ) |  | throws IOException |

static

---

The documentation for this class was generated from the following file:

- java/broad/core/util/ParseGCTFile.java


---

Generated on Fri Aug 15 2014 15:10:57 for ESAT by  

 1.8.7
